# Chromogranin A demonstrates higher expression in preeclamptic placentas than in normal pregnancy

**DOI:** 10.1186/s12884-021-04139-z

**Published:** 2021-10-07

**Authors:** Michalina Bralewska, Lidia Biesiada, Mariusz Grzesiak, Magda Rybak-Krzyszkowska, Hubert Huras, Agnieszka Gach, Tadeusz Pietrucha, Agata Sakowicz

**Affiliations:** 1 Department of Medical Biotechnology, Medical University of Lodz, Zeligowskiego 7/9, Lodz, Poland; 2 Department of Obstetrics, Perinatology and Gynecology, Polish Mother’s Memorial Hospital-Research Institute in Lodz, Rzgowska 281/289, Lodz, Poland; 3 Department of Obstetrics and Perinatology, University Hospital in Krakow, Kopernika 36, Krakow, Poland; 4 Department of Genetics, Polish Mother’s Memorial Hospital-Research Institute in Lodz, Rzgowska 281/289, Lodz, Poland

**Keywords:** Chromogranin a, Catestatin, Preeclampsia, Pregnancy, Placenta, Pregnancy hypertension

## Abstract

**Background:**

Although preeclampsia has long been recognized as a condition affecting late pregnancy, little is known of its pathogenesis or treatment. The placenta releases a number of hormones and molecules that influence the course of pregnancy, one of which is chromogranin A, a soluble protein secreted mainly from the chromaffin cells of the adrenal medulla. Its role in pregnancy and pregnancy-related disorders remains unclear. Therefore, the main aim of the proposed study is to determine whether chromogranin A is related with the occurrence of preeclampsia.

**Methods:**

Placental samples were collected from 102 preeclamptic patients and 103 healthy controls, and Chromogranin A gene (CHGA) expression was measured using real-time RT-PCR, The RT-PCR results were verified on the protein level using ELISA. The normal distribution of the data was tested using the Shapiro-Wilk test. The clinical and personal characteristics of the groups were compared using the Student’s t-test for normally-distributed data, and the χ^2^ test for categorical variables. The Mann-Whitney *U* test was used for non-normally distributed data. As the log- transformation was not suitable for the given outcomes, the Box- Cox Transformation was used to normalize data from ELISA tests and CHGA expression. Values of *P* < .05 were considered statistically significant.

**Results:**

Chromogranin A gene expression was found to be significantly higher in the study group than in controls. Protein analyses showed that although the CgA concentration in placental samples did not differ significantly, the catestatin (CST) level was significantly lower in samples obtained from women with preeclampsia, according to the controls.

**Conclusions for practice:**

This study for the first time reveals that chromogranin A gene expression level is associated with preeclampsia. Moreover, the depletion in catestatin level, which plays a protective role in hypertension development, might be a marker of developing preeclampsia. Further studies may unravel role of Chromogranin A in the discussed disease.

## Background

Preeclampsia (PE) is a multifactorial, hypertensive disorder that affects 2–8% of pregnancies and poses a serious threat to mother and foetus [1]. Among the risk factors the most common mentioned are as follows: family history of PE, maternal age higher than 40, symptoms of hypertension before pregnancy, gestational diabetes mellitus or maternal immunological disorders, multifetuses pregnancy and male sex of the fetus [2, 3]Although our understanding of the risk factors of PE and its molecular foundations is constantly growing, its exact mechanism remains unknown. The most widely-accepted theory proposes that the condition derives from the placenta; it is believed that an abnormally-formed, shallow placenta commences hypoperfusion, followed by hypoxia and placental apoptosis. Although numerous molecular pathways are thought to play a role in the observed changes, such as oxidative stress, endothelial dysfunction, angiogenic imbalance or impaired immune tolerance, none of them fully account for the onset of PE.

PE is a pathological condition that is unique to pregnancy. As such, it pathogenesis has both a molecular and an endocrine basis, and any study must also consider its pregnancy-related adaptations. The placenta, as an endocrine organ, produces and releases hormones and active molecules with a direct impact on the course of pregnancy. Of these, progesterone, oestrogens, human placental lactogen (hPL), human chorionic gonadotropin (hCG) and leptin have the greatest influence and have been most widely studied [4, 5]. In contrast, little data exists regarding the hormone chromogranin A (CgA), a soluble protein secreted mainly from the chromaffin cells of the adrenal medulla, although also found to be produced by the placenta [6]. CgA itself has been implicated in numerous physiological processes: it is believed to play a role in secretory granule biogenesis, support immune activity, serve as a valid neuroendocrine marker, and to influence the course of cardiovascular disorders such as hypertension [7–9]. More importantly, CgA derivedpeptide, catestatin (CST), is believed to play a key role in blood pressure regulation [7, 10, 11]. Hence, as with other placental hormones, CgA may have a significant impact on pregnancy and its hypertensive complications.

The aim of the present study was to identify possible differences between the level of CgA expression and protein concentration in preeclamptic and healthy placentas. Moreover, the difference in the placental concentration of CST, being one of the most important regulating blood pressure factors, between preeclamptic and normotensive pregnancies was examined. Because the sex of the foetus is one of the risk factors of PE development the obtained results were also analysed in the context of fetal-sex-specific pathomechanism of this disease.

## Methods

### Patient selection and data collection

In total, 102 preeclamptic cases and 103 controls were recruited to the study. Written informed consent was obtained from all participants before the delivery. The study protocol was approved by the Medical University of Lodz Ethical Committee. The study population included singleton pregnancies complicated by PE, which was diagnosed according to the following criteria: hypertension (systolic blood pressure over 140 mmHg and diastolic blood pressure over 90 mmHg; measured twice with an interval of at least 6 h) and proteinuria (above 300 mg/24 h or at least 2 + on a dipstick). Both symptoms appeared after 20 weeks of gestational age in previously normotensive women. For the study group were enrolled women with early PE (<34th week of gestation) as well as mothers developing the symptoms of PE at, or after 34 week of gestation (late PE). All women qualified for the study group later delivered by caesarean section. The control group comprised volunteers without PE with singleton normotensive pregnancies [12].

The further following exclusion criteria were used for both the preeclamptic and control groups: chronic diseases, maternal body mass index (BMI) before pregnancy > 30 kg/m^2^ or foetal chromosomal abnormalities. To eliminate any potential influence associated with the form of delivery, placentas in the control group were obtained only from women who delivered by caesarean section without prior contractions. The caesarean section in the control group was conducted due to the following criteria: transverse or breech position of the foetus, ophthalmological indications, orthopaedic indications, or an increased risk of uterine rupture due to a previously performed caesarean section.

Placental fragments of about 2 cm^3^ were taken immediately after birth. The fragments were trimmed approximately 5 cm from the site of the umbilical cord insertion into the placenta. The decidua and amnion were removed. Following the drainage of excess blood, the placental samples were immediately washed in sterile phosphate buffered saline (PBS) and set in RNA *later* (Ambion) to protect RNA and protein from degradation. The tissues were portioned and stored at − 20 °C until further analyses [12].

### RNA isolation and cDNA synthesis

Total RNA was isolated from placentas using the Total RNA Mini Kit (A&A Biotechnology, Poland), according to the manufacturer’s protocols. The RNA quality and purity of the samples were determined spectrophotometrically (NanoDrop, Thermo Fisher Scientific, Poland). The samples were qualified for further analysis when the OD 260/280 ratio was between 1.8 and 2.0. The reverse transcription of 2000 ng of pure RNA into cDNA was conducted by the Maxima First Strand cDNA kit (Thermo Fisher Scientific, Poland) according to the manufacturer’s instructions. The obtained cDNA was stored at − 20 °C for further analysis [12].

### Gene expression analysis

Chromogranin A gene (CHGA) expression in the placenta was analysed by RT-PCR in real time using primers obtained from RT^2^ qPCR Primer Assay (200), (Qiagen; RefSeq Accession no.: NM_001275.3). The procedure was performed using RT^2^ SYBR Green Mastermix according to the manufacturer’s guidelines: RT^2^ SYBR Green Mastermix was combined with Qiagen CHGA primers and aliquated into the 96-well plate. Subsequently, proper amount of the cDNA of each sample, obtained in the previous step (2.2), was added into the wells RT-PCR was performed in a LightCycler 480 (Roche, Mannheim, Germany) device. The stability of the housekeeping gene candidates was analysed using Norm Finder software [13]. Each run was normalized using reference sample prepared from 160 pooled randomly-selected samples of first-strand cDNA from the study (80 samples) and control (80 samples) groups in equal volumes. The normalized gene expression was calculated according to Pfaffl [14].

### Protein isolation

Placental tissue fragments, of approximately 0.1 mg, were washed in PBS and homogenized using 1000 μL of PBS pH 7.2 supplemented by 1 × protease and phosphatase inhibitor cocktail. The homogenates passed through three freeze–thaw cycles comprising freezing at− 80 °C for 10 min followed by thawing. The samples were then passed through a 26-gauge needle 10 times and centrifuged at 14,000 rpm for 10 min at 4 °C. Following the centrifugation, the rich total protein fraction supernatant was collected. The concentration of the placental total protein fraction was detected using the BCA method (Pierce™ BCA Protein Assay Kit, Thermo). The protein fraction was divided into small portions, and each was stored at− 80 °C for future analyses [12].

### ELISA analyses

Quantitative determination of the CgA and CST proteins in the placental total protein fractions was performed using Enzyme Linked-Immunosorbent Assay (ELISA). All procedures related to ELISA were performed according to the manufacturer’s instructions (CloudClone, Wuhan, China for CgA, and RayBiotech, USA for CST). The dilution factors for each analysed protein were experimentally estimated as follows: 100-fold for CgA and 2-fold for CST (the CST dilution was forced by the ELISA protocol, no dilution was made prior to the test performance). The ELISA results were recalculated accordingly: nanograms (ng) or picograms (pg) of studied protein per 100 μg of total protein isolated from the placental sample. Each sample was analysed in duplicate.

### Statistical analyses

All data were analysed using Statistica software version 13.1 (StatSoft, Krakow, Poland). The normal distribution of the data was tested using the Shapiro-Wilk test. The clinical and personal characteristics of the groups were compared using the Student’s t-test for normally-distributed data, and the χ^2^ test for categorical variables. The Mann-Whitney *U* test was used for non-normally distributed data. As the log- transformation was not suitable for the given outcomes, the Box- Cox Transformation was used to normalize data from ELISA tests and CHGA expression. The correlations between the numerical data were analyzed by the Spearman rank correlation coefficient test. Values of *P* < .05 were considered statistically significant.

## Results

### Characterization of study population

Clinical details of the study are given in Table 1. Newborn weight, height,achieved Apgar score and gestational age, maternal platelet number and mean corpuscular volume were lower in the group of women with PE than in healthy controls. The study group displayed significantly higher BMI values and higher numbers of primiparas.

**Table 1:** Comparison of clinical data within the study population

Clinical data	Preeclamptic group (***n*** = 102)	Control group (***n*** = 103)	***P*** value
Age of the women, y^a^	30.9 ± 5.8	32.2 ± 4.5	0.06
WBC, 10^3^/μL^a^	10.4 ± 2.4	10.6 ± 2.2	0.41
RBC, 10^6^/μL^d^	4.1 (3.7–4.5)	4.24.0–4.4)	0.33
HB, g/dL^a^	12.4 ± 2.2	12.4 ± 1.1	0.98
HCT, %^a^	35.5 ± 3.4	36.2 ± 2.7	0.11
MCV, fL^a^	85.3 ± 6.4	87.0 ± 5.1	0.04^c^
MCHC, g/dL^a^	34.3 ± 2.1	33.8 ± 1.6	0.08
PLT, 10^3^/μL^a^	198.5 ± 64.6	223.7 ± 55.5	0.00^c^
BMI, kg/m^2a^	26.4 ± 3.1	24.7 ± 3.6	0.00^c^
Week of gestation Early PE (*n* = 39) vs controls	32 (30–33)	39 (38–39)	0.00 ^c^
Week of gestation Late PE (*n* = 63) vs controls	38 (37–39)	39 (38–39)	0.01 ^c^
Weight of the newborn, g^a^	2409.9 ± 938.3	3409.2 ± 433.0	0.00^c^
Height of the newborn, cm^a^	48.3 ± 6.6	54.5 ± 2.8	0.00^c^
Apgar score, 1 min^a^	8.6 ± 1.6	9.5 ± 0.8	0.00^c^
Primiparas, n %^b^	69 (68%)	37 (36%)	0.00^c^
Miscarriages, n %^b^	19 (19%)	21 (20%)	0.75
Baby sex (male), n %^b^	60 (59%)	47 (46%)	0.06

*HB* haemoglobin, *HCT* haematocrit, *MCV* mean corpuscular volume, *MCHC* mean corpuscular haemoglobin concentration, *PLT* platelet count, *RBC* red blood count, *WBC* white blood count

^a^The values are presented as mean ± SD. Analysis was performed using Student’s t-test

^b^Analysis was performed using the χ^2^ test

^c^Denotes statistical significance. *p* < 0.05 was considered as significant

^d^The values are presented as median with interquartile range. Analysis was performed using the Mann-Whitney *U*test

### Preeclamptic placentas differ in the expression level of Chromogranin A from non-complicated cases

**Fig. 1:**
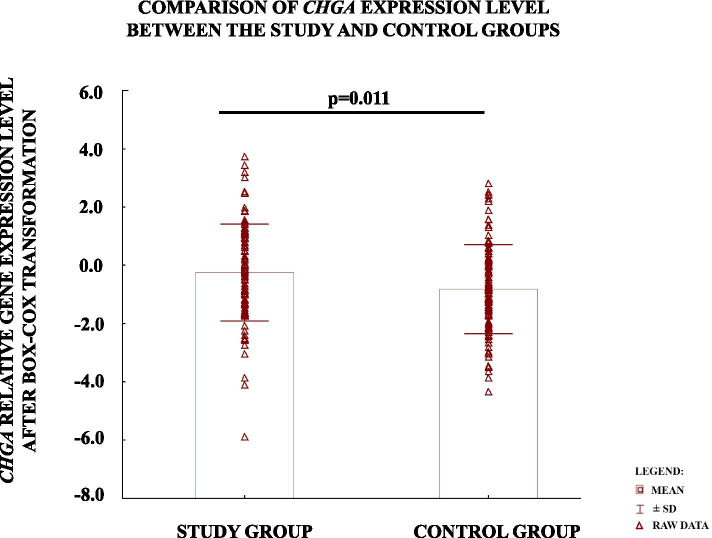
Comparison of relative *CHGA* gene expression level between study and control group. The values are presented as mean ± SD after Box-Cox Transformation. *p*-vaule was calculated using the Student’s t-test

The level of CHGA gene expression in preeclamptic placentas (− 0.25 ± 1.7) was found to be significantly higher than in controls (− 0.82 ± 1.5) (Fig.1).

**Fig. 2:**
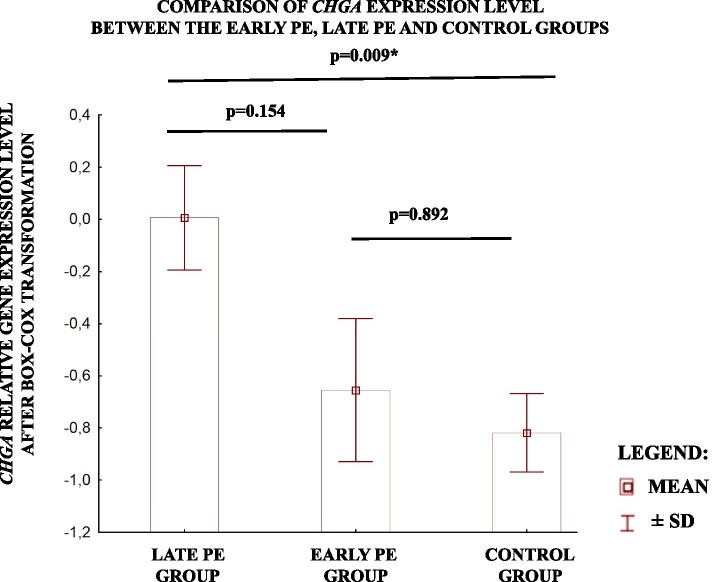
Comparison of relative *CHGA* gene expression level between study groups divided to early and late PE, and control group. The values are presented as mean ± SD after Box-Cox Transformation. *p*- value was calculated using One-way ANOVA with Turkey’s post hoc test for various number of cases in each group * Denotes statistical significance

Furthermore, after the division of the study group into the late and early PE, the level of *CHGA* gene expression was found to be significantly higher in late PE group in comparison to healthy controls. No statistically significant results were obtained for comparison of *CHGA* gene expression level between early PE and control groups (Fig.2).

Moreover, the possible relationships between CHGA gene expression level and maternal or fetal clinical features were examined. There was no observable correlation between maternal BMI and *CHGA* gene expression level (R_Spearman_ = − 0.016; *p* = 0.817). Additionally, the *CHGA* gene expression level did not differ between primiparous and multiparous mothers (− 0.43 ± 1.59 vs − 0.63 ± 1.64; *p* = 0.369). Similar, insignificant results were obtained for comparison of *CHGA* gene expression level and the sex of the foetus (− 0.38 ± 1.58 for male vs − 0.69 ± 1.64 for female; *p* = 0.178).

### The protein level of CST was lower, whereas CgA did not show a significant difference in Preeclamptic placentas

The mass fraction assessments of CgA and CST proteins were presented in the Table 2.

**Table 2:** Comparison of the mass fraction assessment of CgA and CST proteins between the study and control group

Mass fraction^*^	Preeclamptic group mean ± SD	Control group mean ± SD	***P*** value^b^
CgA protein^**a^	23.3 ± 3.0	23.4 ± 2.7	0.69
CST protein^**a^	6.4 ± 1.0	6.7 ± 1.4	0.04

^*^The mean and standard deviation (SD) were calculated for the result of mass fraction assessment as follows: ^**^CgA [pg] per 100μg of total protein fraction isolated from placenta or CST [ng] per100 μg of total protein isolated from placenta

^a^analysis was performed using Box-Cox Transformation

^b^*p*-value calculated using Student’s t-test; *p* < 0.05 was considered as significant

After Box-Cox transformation the mean ± SD values of the CST mass fraction for the study group were significantly lower than in the healthy controls. In the same time, there was no observable significant difference in the CgA protein level between the studied groups (Table 2).

This study did not show any correlation between the studied proteins i.e. CgA and CST both in studied and control placentas (R_Spearman_ = − 0.139, *p* = 0.163 and R_Spearman_ = − 0.014, *p* = 0.898, respectively). However, the weak negative correlation between *CHGA* gene expression level and CgA protein concentration for control placentas was observed (R_Spearman_ = − 0.243, *p* = 0.017).

**Fig. 3:**
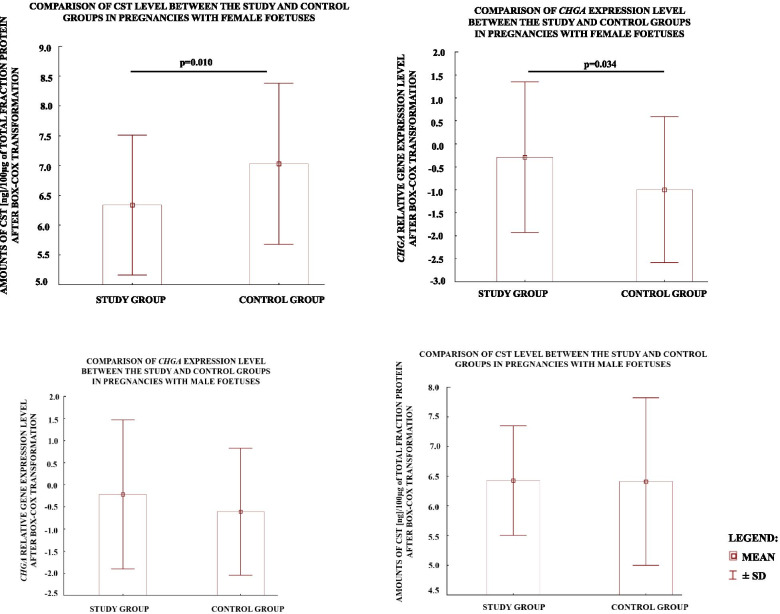
Comparison of the CST level (**a, c**) and *CHGA* expression level (**b, d**) between the study and control groups in pregnancies with female (**a, b**) and male foetuses (**c,d**). The values are presented as mean ± SD after Box-Cox Transformation. *p*-value was calculated using the Student’s t-test

As the male sex is one of most common mentioned risk factor of preeclampsia development, we examined whether the gene expression and protein level of chromogranin A, and its derived peptide i.e. catestatin, might differ between preeclamptic and normotensive placentas bearing only male or only female foetus. Interestingly, CST protein level was significantly lower in preeclamptic pregnancies with female foetuses than in corresponding controls (Fig. 3a), whereas the mean ± SD values of the *CHGA* expression level presented an opposite outcome, showing higher *CHGA* expression in pregnancies with female foetuses than in corresponding controls (Fig. 3b). No relationship was found between the CST protein level or CHGA expression and male sex of the foetus (Fig. 3c and d). This suggests that patomechanism of preeclampsia may differ between pregnancies bearing male and female foetuses and chromogranin A, and its derived peptide might be implicated in the patomechanism of preeclampsia induced by female foetus.

## Discussion

The aim of the present study was to examine whether CgA, or its derived peptide CST, are implicated in the complex process of PE pathogenesis. Our findings not only confirm presence of CgA in human placenta, but also gave the first evidence that the level of expression of the CHGA gene in the placenta differs between healthy pregnancies and preeclamptic ones. Furthermore, the present article is the first to examine the possible role of CgA in PE. Until now, only a few studies investigated the role of CgA in pregnancy, however none of these studies focused on PE or pregnancy hypertension [15, 16].

Human CgA is a 49 kDa acidic polypeptide belonging to the granin family. Proteolytic cleavage of CgA results in the production of many biologically-active peptides. The main functions of CgA are the biogenesis, storage, trafficking and release of catecholamines from the dense core secretory granules of adrenergic neurons [17]. In addition, each of the CgA-derived peptides has its own molecular function, which becomes apparent depending on the local microenvironment, thus to understand the role played by CgA in pregnancy and PE, it is first necessary to determine the local differences in its processing and function, as well as get acquainted with PE molecular basis.

During pregnancy, the exchange of nutrients, oxygen and waste products between mother and fetus depends on the correct formation of highly-resistant, large vessels. In PE, this process is impaired and the resulting small caliber spiral arteries are not sufficient to fulfill the growing demands of the developing foetus. The reduced blood flow and prolonged state of placental ischaemia occurring as a result lead to the development of hypoxia. The poorly-perfused placenta starts to produce reactive oxygen species (ROS), pro-inflammatory cytokines and autoantibodies, thus inducing vasoconstriction and exacerbating maternal endothelium dysfunction [18, 19].

It is obvious that placenta, which is a unique endocrine organ of pregnancy, produces and releases hormones into the maternal blood circulation. One of those hormones is CgA, which is known to be implicated in a blood pressure regulation [20]. Above can be confirmed by the fact, that in the hypertensive pheochromacytoma patients, CgA concentrations are used as a diagnostic marker [21]. Furthermore, it is known that in this group of patients antihypertensive drugs are used to manage hypertension, to control associated cardiovascular symptoms, and to prepare patients for operation [22]. However, the only effective cure is a resection of the cancer [23]. This way of treatment stands as a common feature of neoplasm cancer and PE, as the only known cure for the preeclamptic mother is a premature caesarian section [24].

In the present study *CHGA* gene expression level was higher in PE patients than in the healthy controls. What is more, after the study group division into the early and late PE, in the late PE group the *CHGA* gene expression level was significantly higher than in control group. In the same time, there was no observable difference between early PE and control groups. Presented outcomes indicate that *CHGA* gene expression level in human placenta is not influenced by the gestational age, but more likely is the effect of the ongoing pathological process. The process seems to be especially important for the late PE cases.

Interestingly, the negative correlation found between the gene expression level coding for CgA and the CgA protein characterizes only the control placentas; the lower level of CHGA expression allow for high CgA level in placental cells. This might be related with the high efficiency of post-translational processes influencing the CgA level in the cells, which is necessary for production of small peptides being a product of its cutting. These small peptides, especially catestatin, are strongly implicated in the regulation of maternal blood tension [25]. Interestingly, our study presented that this peptide is depleted in preeclamptic placentas. We believe that these posttranscriptional mechanisms are disturbed in preeclamptic placentas leading to the lack of balance between the CHGA expression, chromogranin A level and small peptides being the result of its cut.

According to the literature, CgA is involved in the regulation of majority of the processes ongoing in PE. In vitro studies on human umbilical vein endothelial cells (HUVECs) indicate that the N- terminal fragment of CgA, vasostatin I (VS-1), inhibits the activation of hypoxia-triggered hypoxia-inducible factor 1 (HIF-1α) by preventing its nuclear translocation [26]. During PE development HIF-1α is abnormally elevated, influencing placentation and placental function [18, 27]. Among others, it regulates levels of plasminogen activator inhibitor type 1 (PAI-1) in cytotrophoblasts, also found to be elevated in PE. PAI-1, in the other hand, inhibits the activity of tissue plasminogen activator (tPA). Diminished tPA prevents conversion of plasminogen to plasmin, an enzyme implicated in CgA proteolitic clavege, with production of peptide called CST [28]. In PE levels of both, tPA and PAI-1 are altered, thus affecting the placentation process and possibly influencing the disease progression [29, 30].

Mahapatra et.al, conducted an in vivo study in which targeted ablation of CgA resulted among others in elevation of blood pressure [31]. In the other experiment, Gayen et al. tested CHGA gene knockout mice (*Chga−/−)* obtaining similar results of elevated systolic and diastolic blood pressure in the control group. Furthermore, *Chga−/−* mice exhibit increased ROS, isoprostane levels (pointing at lipid peroxidation) and depleted endothelium- derived relaxing factor, nitric oxide (NO), all of which are known to be implicated in the oxidative stress developing in PE [18, 32]. In the other hand, in hypertensive patients there is an observable increase in plasma CgA level, which also displays substantial heritability [8]. This apparent contradiction can be explained by the variety of biological activities of CgA-derived peptides. One of the mostly studied among them CST. CST is a 21- amino acid residue peptide, a key factor influencing many of the characteristics of PE, including hypertension, inflammation, vasodilatation and angiogenesis [10, 33–36]. CST level is known to be diminished in hypertensive patients, which was also confirmed by the in vivo studies on CST knockout mice (CST-KO) developing hypertension [37–39]. Furthermore, clinical studies on healthy and hypertensive patients revealed its dose-dependent vasodilatory, thus protective effect [40]. Presented literature data stays with the agreement with our results, in which despite the high CHGA expression, CgA protein did not differ among the groups, while CST peptide concentration was diminished in the study group. According to the given outcomes, there is a probability that protective CST peptide, in PE patients, is released in smaller amounts into the maternal blood circulation. Further studies assessing CST concentration are necessary to provide more data and to confirm our theory.

What is more interesting, concerning our outcomes, according to the experiment conducted by the Fung M. et al., healthy female subjects have higher plasma CST level, than male, yet lower CgA precursor [40]. Above was explained by the increased processing of CgA to CST in females. In our study, the sex of the foetus influenced the levels of both CgA and CST in placental cells. It is known that pregnancies bearing male foetuses predispose to occurrence of preeclampsia [3]. It could be related with the hormonal status; placental cells secrete to the maternal blood a lot of hormones which depend form the pregnancies bearing male and female foetuses [41]. The present study has shown that both CgA and CST levels differ significantly between placentas bearing female foetuses obtained from preeclamptic and normotensive pregnancies. This suggests that chromogranin A may be implicated in the fetal-sex-specific pathomechanism of preeclampsia. The further studies are necessary to examine the association between hormonal status and chromogranin A action in the context of preeclampsia.

Apart from the discussed issues, CgA and CST are also implicated in regulation of, observed in PE, chronic inflammation. In this state, placenta releases various pro-inflammatory cytokines, including interferon γ (IFNγ), tumor necrosis factor α (TNFα), interleukin 6 (IL-6), interleukin 1 β (IL-1β), as well as other molecules likenuclear factor-κB (NF-κB) [42–46]. TNFα initiation of inflammation is mainly mediated by the activity of intercellular adhesion molecule 1 (ICAM-1), which allows leucocytes to be attached to the endothelium and increases vascular permeability [44]. Treatment with CgA-containing sera resulted in significant inhibition of TNFα-elicited ICAM-1 up-regulation by endothelial cells [17]; similarly, the CgA peptide, VS-1inhibited TNFα-mediated gap formation and vascular leakage in cultured bovine pulmonary and coronary arterial endothelial cells [47]. TNFα and IFNγ injection augmented CgA protein level in the colonic mucosa of mice treated in vivo ^42^. Interestingly, another pro-inflammatory marker, Il-6, was found to significantly increase the expression of CHGA in prostate cancer cells [48]. Although it is presently unknown why CgA levels increase after stimulation with pro-inflammatory cytokines, recent findings indicate that CgA-derived CST exerts an anti-inflammatory effect by modulating pro-inflammatory and anti-inflammatory cytokine expression [36, 49].

An additional key component of the immune response is nuclear factor-κB (NF-κB). Its elevated activation is characteristic of PE, during which it further stimulates the transcription of IL-1β, a pro-inflammatory cytokine [45, 46]. Microglia have been found to exhibit both increased NF-κB activation and IL-1β production following treatment with CgA [50]; however, a CgA fragment belonging to VS-I was found to elicit an anti-inflammatory effect and inhibit NF-κB in vitro in a human monocyte cell line [51]. These significant discrepancies between the two studies may derive from differences in the choice of starting material and studied protein fragments.

## Conclusions

In the present study, the expression of the CHGA in the preeclamptic placentas was significantly higher than in the placentas from healthy patients, whereas CST protein level was diminished in the study group. Our findings, and those of previous studies, suggest that during PE the placenta produces and releases larger amounts of CgA hormone in order to protect the developing fetus and to maintain pregnancy. Special concern should be paid to CgA-derived peptides, in particular to CST. There is a possibility that local processing of CgA may result in the inhibition of inflammatory processes and prevent hypoxia-mediated hypertension and proteinuria. In addition, locally present molecules, such as PAI-I, may modulate the final effect of CgA on the course of pregnancy by influencing production of its derived peptides. The major limitations of the present study comprise lacking measurements of CgA and CST concentrations in blood of the mothers. Furthermore, in vitro studies on trophoblast cell lines are necessary to establish whether the cells are able to secrete CgA derived peptides. Consequently, further studies covering the discussed area are urgently required as the possible outcomes will serve as a step towards determination of the role of CgA in PE and pregnancy.

## Data Availability

All data generated or analyzed during this study are included in this published article.

## Abbreviations

BMI: Body mass index
CgA: Chromogranin A
CHGA: Chromogranin A gene
Chga−/−: Chga gene knockout mice
CST: Catestatin
CST-KO: CST knockout mice
HB: Haemoglobin
hCG: Human chorionic gonadotropin
HCT: Haematocrit
HIF-1α: Hypoxia-inducible factor 1
hPL: Human placental lactogen
HUVECs: Human umbilical vein endothelial cells
ICAM-1: Intercellular adhesion molecule 1
IFNγ: Interferon γ
IL: Interleukin
MCHC: Mean corpuscular haemoglobin concentration
MCV: Mean corpuscular volume
NF-κB: nuclear factor-κB
NO: Nitric oxide
PAI-1: Plasminogen activator inhibitor type 1
PBS: Phosphate buffered saline
PE: Preeclampsia
PLT: Platelet count
RBC: Red blood count
ROS: Reactive oxygen species
TNFα: Tumor necrosis factor α
tPA: Tissue plasminogen activator
VS-1: Vasostatin I
WBC: White blood count
